# Shoe heel abrasion and its possible biomechanical cause: a transversal study with infantry recruits

**DOI:** 10.1186/s13018-015-0319-0

**Published:** 2015-11-19

**Authors:** Daniel Baumfeld, Fernando C. Raduan, Benjamim Macedo, Thiago Alexandre Alves Silva, Tiago Baumfeld, Danilo Fabrino Favato, Marco Antonio Percope de Andrade, Caio Nery

**Affiliations:** UFMG—Federal University of Minas Gerais, Juvenal dos santos St, 325, 30380 5030 Belo Horizonte, MG Brazil; Foot and Ankle Clinic, UNIFESP, Escola Paulista de Medicina, São Paulo, SP Brazil; Felicio Rocho Hospital, Belo Horizonte, MG Brazil; Madre Teresa Hospital, Belo Horizonte, MG Brazil

**Keywords:** Infantry recruits, Shoe-heel abrasion, Ankle sprains, Calf muscle shortening, Foot alignment

## Abstract

**Background:**

Excessive shoe heel abrasion is of concern to patients and shoe manufacturers, but little scientific information is available about this feature and its possible causes. The purpose of this study was to relate this phenomenon with biomechanical factors that could predispose to shoe heel abrasion.

**Methods:**

Ninety-seven recruits (median age 25) were enrolled in this study. Shoe abrasion was assessed manually with a metric plastic tape on the posterior part of the heel that comes in contact with the ground. The number of sprains, foot alignment, and calf muscle shortening (*Silfverskiold* test) was also assessed in order to relate it with shoe heel abrasion. After using our exclusion criteria, 86 recruits and 172 were considered for this study.

**Results:**

The most common abrasion site was the lateral portion of the heel surface (50 %). Forty-four percent of the participants had neutral hind-foot alignment and 39 % had valgus alignment. Twenty-six (30 %) patients have had previous ankle or foot sprains. Neutral foot was related with less calf muscle shortening. On the other hand, valgus hind-foot alignment was more associated with Achilles shortening (*p* < 0.05). Patients with neutral alignment were associated with more uniform shoe heel abrasion and varus feet were associated with more central and lateral abrasion (*p* < 0.05). The pattern of shoe heel abrasion was not statistically related with calf muscle shortening nor with number of sprains.

**Conclusion:**

This study was able to correlate shoe heel abrasion with biomechanical causes (neutral alignment–uniform abrasion/varus alignment–central and lateral abrasion). More effort has to be done to continue evaluating outsole abrasion with its possible biomechanical cause in order to predict and treat possible associated injuries.

## Introduction

Excessive shoe heel abrasion is of concern to patients and shoe manufacturers, but little scientific information is available about this feature and its causes. There is no established relationship between shoe heel abrasion and biomechanical or anatomical causes nor with any potential harm to patients [1].

In normal gait, from heel strike to toe-off, floor reactions, joint motions, and muscle activity change constantly. These determine the normal centers of pressure in the feet during the walking cycle.

Because of the normal 15° (13°–25°) [2] of external rotation *Kite’s* walking angle, the center of pressure (COP) starts its path at the sole of the foot by the posterolateral border of the hind-foot during the heel strike; soon it reaches the center of the calcaneal and progresses forward very quickly through the mid-foot in a straight line directed to the center of the ball of the forefoot (between the second and third metatarsal heads). At this point, the speed of dislocation of the COP decreases dramatically while the hind-foot leaves the ground. After some milliseconds, the COP progresses to the toes, where the toe-off begins [3]. Normal external rotation of the walking angle can be explained by the external rotation of the ankle joint axis, the oblique metatarsal break, and the action of the plantar aponeurosis after heel rise begins [4].

The expected progression of the COP as well as the physiologic action of the midtarsal and tarsometatarsal joints can be affected by many pathologies of the foot as cavovarus and planovalgus feet, hyperpronated foot, tarsal coalition, and paralytic and insensitive foot [5].

It is described that infantry boots may be related to overuse injuries in recruits [1, 6], and these kinds of shoe wear must be well studied to understand why it happens. Finestone et al. demonstrated the amount of abrasion in recruits’ boots that may predispose to ankle sprains, an injury that should be consistently avoided in this population [1]. Bohm et al. also described that increased boot shaft stiffness decreases ankle room of motion, decreasing gait efficiency and overloading knee and hip joints. This is an important factor that could predispose recruits to muscle fatigue and injuries during walking or hiking [7].

An interesting study tried to access the influence of asymmetric shoe wear abrasion on lower limb performance. They did found a lower performance on single leg heel raise task when the medial column was higher than the lateral [8]. This implies that outsole abrasion could affect recruits’ performance and reinforces the need to study the abrasion patterns.

Some authors have tried to correlate the thickness of the sole with joint position sense, what could change during the years of shoe abrasion, but they were not able to correlate a meaningful change in the joint position sense with different soles [9].

The purposes of this study are to identify, typify, and measure the shoe heel abrasion in a group of infantry recruits and try to correlate it with calf muscle shortening, ankle sprains, and hind-foot alignment.

## Method

This is a transversal study with 97 recruits who signed an informed consent, answered a questionnaire, were examined by the same Foot and Ankle Surgeon, and had their boot heel abrasion measured by the same member of the research team. All the participants used their boots only during their working hours. Our institutional ethics committee has approved this study.

Participants who had previous foot and ankle fractures and had boots with less than 3 months of use were excluded from this study.

We applied a self-responding questionnaire as the first step of the study. The recruits were asked about demographic data, how old were the boots, occurrence and incidence of previous foot or ankle sprains, presence of any foot and ankle pain or disease, and if they had a previous fracture in the foot or/and ankle.

**Fig. 1:**
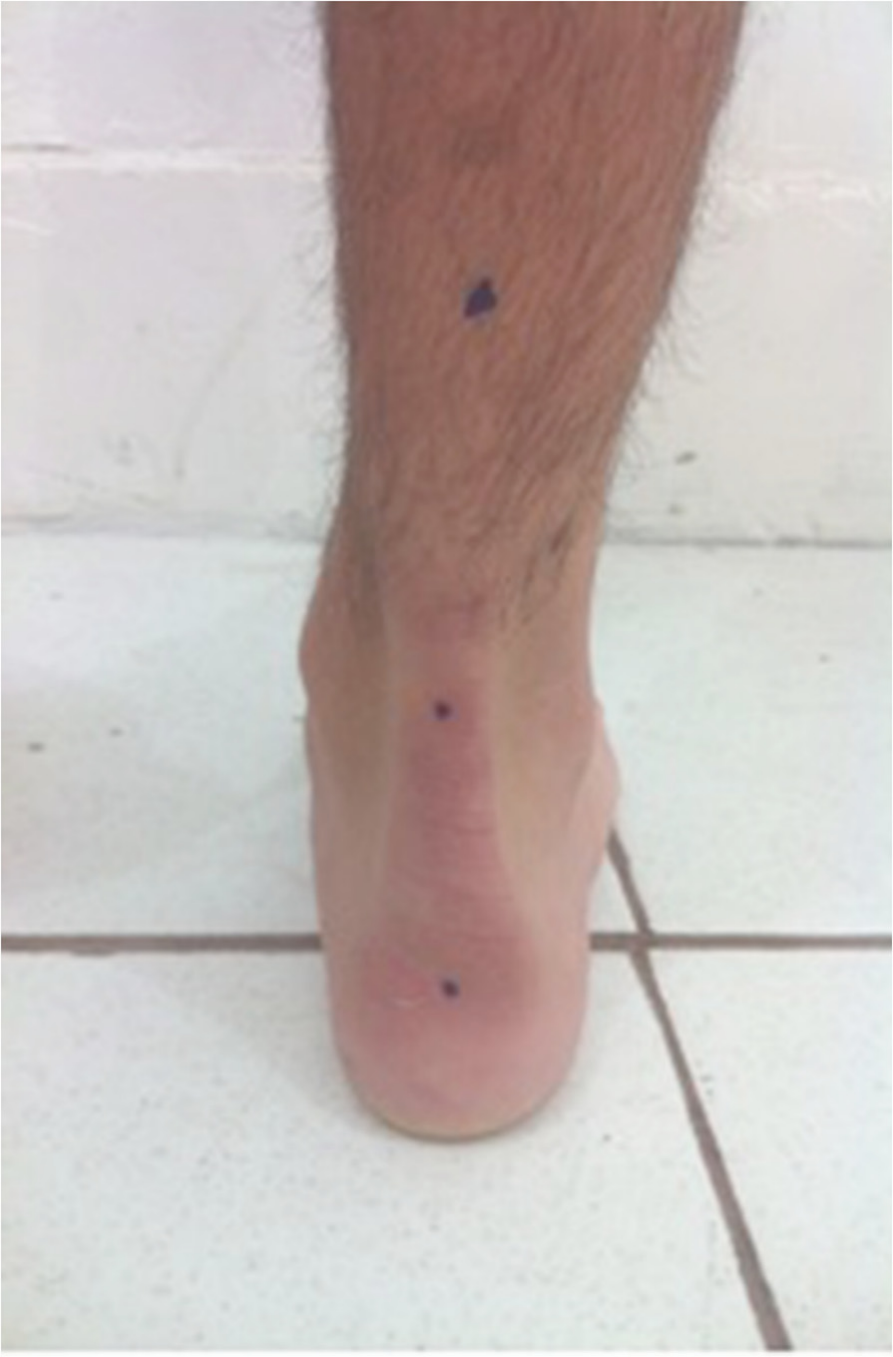
Hind-foot alignment

Foot alignment and deformities were accessed. The posterior alignment of the Achilles tendon and the calcaneus was used to determine the hind-foot characteristic (varus, valgus, or neutral) (Fig. 1). We measured the angle of deformity of the hind-foot with a goniometer, using the points shown in the figure as reference. As none of the patients enrolled in this study had considerable hind-foot deformity but only a different hind-foot alignment of a normal foot, they were considered valgus those with 1°–5° of valgus, neutral those with straight alignment, and varus those with 1°–5° of varus. Calf muscle shortening was also evaluated with the *Silfverskiold* test [10]. Those patients with more than 5° of dorsiflexion with knee extended and flexed were considered with no calf muscle shortening, and those with calf shortening (less than 5° of dorsiflexion) were differentiated in gastrocnemius shortening or Achilles tendon shortening with this test.

**Fig. 2:**
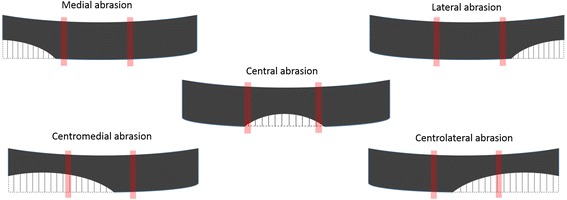
Patterns of shoe heel abrasion

**Fig. 3:**
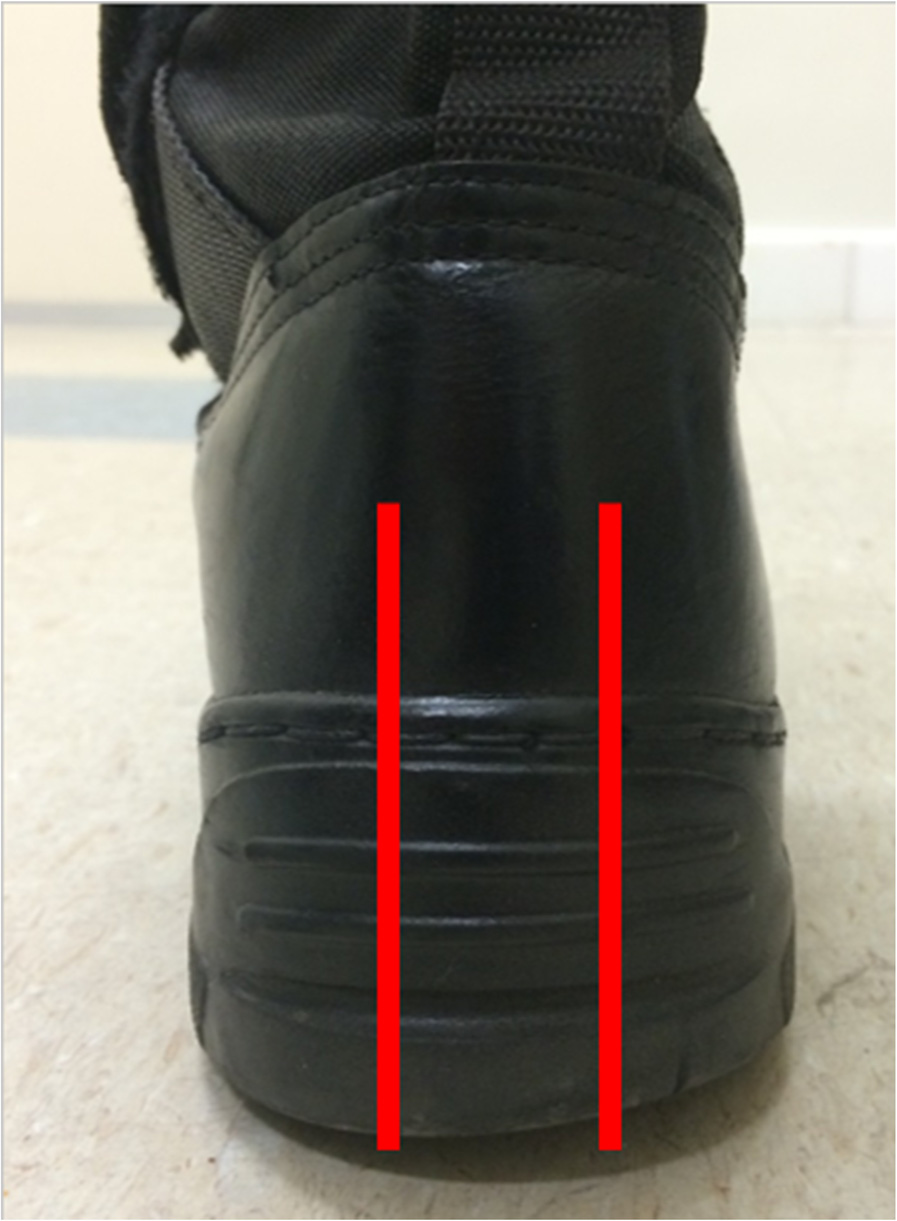
Boot with centrolateral abrasion

Shoe abrasion was assessed manually with a metric plastic tape on the posterior part of the heel that comes in contact with the ground, comparing with a brand new standard army boot (Fig. 2). The posterior part of the heel was divided in three parts using two imaginary longitudinal lines. With this, we could classify abrasion as lateral, medial, or central, if it occurred in one third of the boot. If it occurred in two thirds of the heel, we classified it as centromedial or centrolateral; if it occurred in the three thirds, we classified it as uniform (Fig. 3).

The chi-square test was used in the correlation analysis of foot heel abrasion, foot alignment, sprains, and calf muscle shortening. A 95 % confidence interval was used to conclude that our results were statistically significant (alpha error = 0.05).

## Results

Of the 97 soldiers enrolled in our study, 86 met our inclusion criteria. One was excluded because he had a previous ankle fracture and 10 had new boots. We assumed 3 months as the threshold of our exclusion criteria because all these 10 soldiers did not have any measurable shoe heel abrasion. Therefore, we analyzed 172 feet and boots.

The recruits’ age ranged between 18 and 48 years, weighted between 53 and 100 kg, were between 1.64 and 1.91 m tall and worn boots numbered from 38 to 45 (European) (Table 1). Ninety-five percent were right-handed and 29.8 % have had previous ankle or foot sprains.

**Table 1:** Group description

Variables	Min	Medium	Max
Age (years)	18	25	48
Weight (kg)	53	76	100
Height (m)	1.64	1.76	1.91
BMI	18.38	24.38	32.91
Shoe number	38	41	45
Time since the first use of the shoe (months)	3	14	60

The most common alignment of the hind-foot was neutral, totalizing 79 feet (45.9 %). Sixty-nine (40.1 %) were valgus and 24 (13.9 %) were varus. None of the patients enrolled in this study had considerable hind-foot deformity.

Shoe heel abrasion was most common in the lateral aspect of the boots analyzed (50 %), followed by central abrasion (27.1 %), centrolateral (9.8 %), uniform (8 %), and medial (4.9 %). There was no centromedial abrasion in our study.

About half of the soldiers had negative *Silfverskiold* test (50.5 %), 26 % percent had isolated Achilles contracture, and 22.6 % had gastrocnemius shortening.

Tables 2 and 3 demonstrate our associative analysis. We found that neutral alignment was associated with more uniform shoe heel abrasion and varus was associated with more central and lateral abrasion (*p* < 0.05). We also found that neutral alignment had less calf muscle shortening and valgus is associated with more Achilles contracture (*p* < 0.05).

**Table 2:** Relationship between hind-foot alignment and abrasion pattern

Alignment	Abrasion pattern						
	Lateral	Central	Centrolateral	Uniform	None	Medial	Total
Neutral	40	16	5	12	4	2	79
Valgus	32	18	8	1	5	5	69
Varus	9	10	3	0	1	1	24
Total	81	44	16	13	10	8	172

**Table 3:** Relationship between alignment and posterior chain shortening

Alignment	Posterior chain shortening			
	None	Achilles tendon	Gastrocnemius	Total
Neutral	45	15	19	79
Valgus	26	27	16	69
Varus	16	4	4	24
Total	87	46	39	172

With the numbers available, no significant difference could be detected between shoe heel abrasion and calf muscle shortening nor with previous sprains. Foot alignment was not associated with sprains.

## Discussion

The more common lateral abrasion found on our study may have a physiological biomechanical cause. Because of normal walking progression angle, the first contact of the foot with the ground on heel strike is on the posterolateral border. This first contact may be responsible for great dissipation of energy, thus contributing with significant lateral abrasion [2–4].

Previous studies, also performed with recruits, have described the most common site of abrasion on shoes as posterolateral and they did not found correlation between hind-foot alignment and the predominant local of abrasion [1]. In our study, we found lateral as the most common site of abrasion among soldiers (50 %), and we did found a correlation between foot alignment and abrasion, as neutral alignment predisposed to uniform abrasion and varus alignment predisposed to central and lateral abrasion.

It is well studied that in normal barefoot walking, the forefoot carries a total load three times more than the hind-foot. But in a biomechanical study of the COP, when footwear was worn, the load of the forefoot was progressively reduced as the rigidity of the sole of the shoe increased [11]. This may explain why soldiers, who wear shoes with very rigid sole, have more posterior heel abrasion.

In a study with an addition of varus or valgus wedge inside the shoes, the authors observed that the location of the center of pressure shifts medially with varus wedging and laterally with valgus wedging [12]. Maybe these kinds of insole could change the site of abrasion in infantry recruits but we do not know the benefits of this intervention nor its biomechanical consequences in gait.

In a prospective study of risk factors for Achilles tendinopathy among infantry recruits, 6.8 % were found to have calf muscle shortening [13]. Although we did not evaluate Achilles tendinopathy specifically, our Achilles contracture prevalence was way more common (26.7 %).

Mei-Dan et al. hypothesized that a low arch of the foot might be a risk factor for ankle sprains [14]. Although we did not measure the arch of the foot, valgus alignment of the hind-foot was not related with sprains in our study. Another study, also performed with recruits, stated that taller and heavier individuals were predisposed to ankle sprains [15].

## Conclusion

Based on our results, we can conclude that neutral feet alignment is associated with more uniform shoe heel abrasion and varus feet is associated with more central and lateral abrasion. This is an important finding as it emphasizes the role of the subtalar joint in the distributions of pressure during gait. No relationship was found between shoe heel abrasion and calf muscle shortening nor with previous sprains.

This is the first study to correlate foot heel abrasion with any biomechanical cause, as previous studies were not able to correlate foot alignment with patterns of outsole abrasion (1). More effort has to be done to continue evaluating outsole abrasion with its possible biomechanical cause in order to predict and treat possible associated injuries.
